# The one that takes it all: The essential role of VDAC3 in the redundant control of ABA signaling

**DOI:** 10.1093/plphys/kiad576

**Published:** 2023-10-31

**Authors:** José Manuel Ugalde

**Affiliations:** Assistant Features Editor, Plant Physiology, American Society of Plant Biologists; Institute of Crop Science and Resource Conservation (INRES)-Chemical Signalling, University of Bonn, Friedrich-Ebert-Allee 144, 53113 Bonn, Germany

Gas exchange between plants and their surrounding environment is essential for life on the planet. This gas exchange happens through tiny pores (20–70 *µ*m) on the leaf epidermis called stomata, whose aperture is controlled by a pair of guard cells surrounding the pore (Jezek and Blatt 2017). While stomata opening is crucial for carbon dioxide intake, maintaining an open state exposes the plant to various detrimental effects, such as water loss and a compromised cellular osmotic potential (Ehonen et al. 2019). Controlled stomata closure becomes a necessary adaptation response of plants toward their environment in a process intricately but not exclusively linked to abscisic acid (ABA) and reactive oxygen species (ROS) (Fig. 1A).

**Figure 1.**
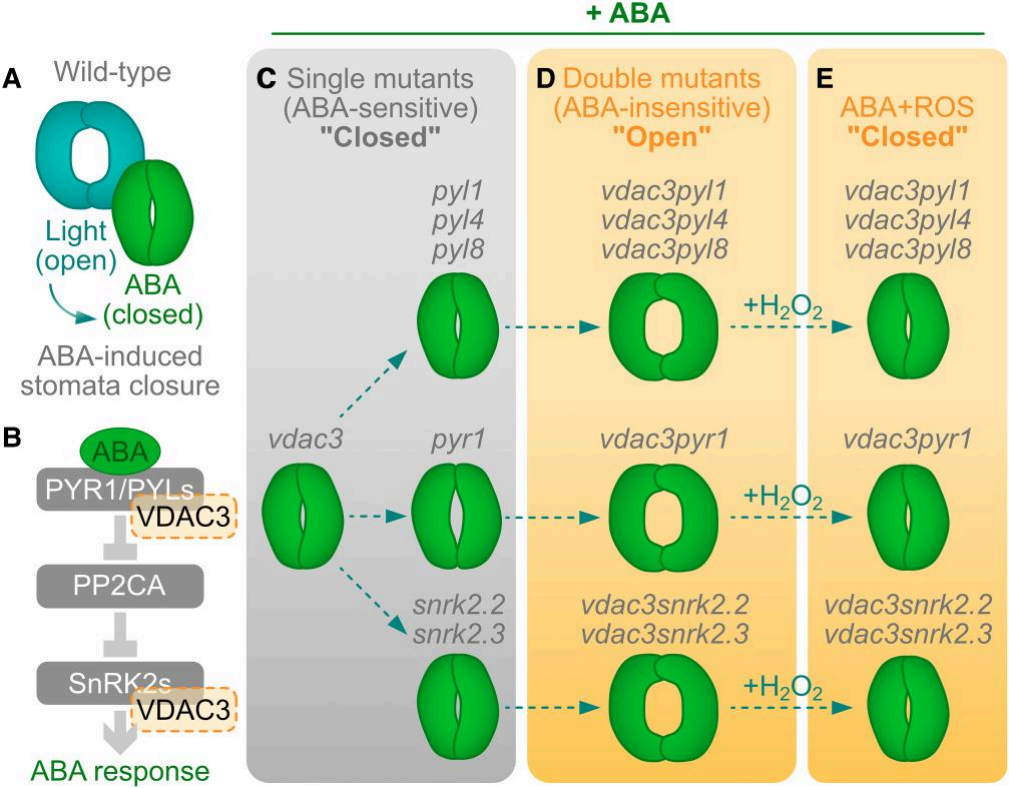
VDAC3 regulates ABA-mediated stomata closure jointly with PYLs PYR1 and SnRK2s. **A)** Stomata opening can be modulated with light to open them or ABA to induce their closure. **B)** The core of ABA signaling includes the receptors PYRABACTIN RESISTANCE1 (PYR1) and the PYR1-like receptor family (PYLs). The ABA-PYL/PYR interaction inhibits the PHOSPHATASE 2CA (PP2CA), which is typically inhibiting SNF1-RELATED PROTEIN KINASES (SnRK2s) responsible for activating ABA-related transcription factors for ABA responses. Here, Qin et al. 2023 postulate that a mitochondrial voltage-dependent anion channel protein, VDAC3, interacts with PYR1, PYLs, and SnRK2s. **C)** Single mutants for VDAC3 (*vdac3*), PYLs (*pyl1, pyl4, pyl8*), PYR1 (*pyr1*), and SnRK2s (*snrk2.2, snrk2.3*) are still sensitive to ABA-induced stomata closure, unlike the double mutant (*vdac3pyl1*, *vdac3pyl4*, *vdac3pyl8*, *vdac3pyr1*, *vdac3snrk2.2*, *vdac3snrk2.3*), which are less susceptible to ABA (**D**). **E)** Exogenous H_2_O_2_ is sufficient to revert the open stomata phenotype in the double mutants. Figure created by J.M.U in Affinity Designer (Version 2.2.0).

In Arabidopsis (*Arabidopsis thaliana*), the ABA signaling core depends on the ABA-receptors PYRABACTIN RESISTANCE1 (PYR1) receptor and the PYR1-LIKE (PYLs) family. Upon interaction with ABA, the PYR/PYL proteins mediate the inhibition of the PROTEIN PHOSPHATASE 2C A (PP2CA), which, under low ABA levels, acts as a negative regulator of SUCROSE NON-FERMENTING 1 (SNF1)-RELATED PROTEIN KINASES (SnRK2s) (Fig. 1B). The SnRK2s are, in turn, positive regulators of the ABA-dependent signal transduction (Hsu et al. 2021). Notably, these signaling components have a high level of functional redundancy when regulating ABA-dependent stomata closure, where high-order mutants such as the sextuple *pyr1pyl12458* are needed to make plants insensitive to ABA-induced stomata closure (Gonzalez-Guzman et al. 2012). Such redundancy is also observed between the kinases SnRK2.2 and SnRK2.3, where only the double mutant between these is partially insensitive to ABA (Fujii et al. 2007).

ABA treatments promote an increment of intracellular ROS levels in guard cells, which have been reported recently to be significant in mitochondria (Postiglione and Muday 2023), an organelle that exhibits important ROS production due to metabolic by-products and fundamental for ABA-mediated stomatal closure (Feitosa-Araujo et al. 2020). In this issue of *Plant Physiology*, Qin et al. show how VDAC3, a mitochondrial voltage-dependent anion channel protein linked to adaptation responses, is an essential component of the redundant PYR/PYL/SnRK2 signaling core for ABA-mediated stomatal closure (Fig. 1A).

The authors proved that VDAC3 interacts with many ABA receptors, such as PYR1, PYL1, PYL2, PYL4, and PYL8, as well as with the kinases SnRK2.2, SnRK2.3, and 2.6 via yeast 2 hybrid, protein pull down, and bimolecular fluorescence complementation assays. The latter approach also helped them elucidate that all these interactions happen in the plant mitochondria. To test the relevance of VDAC3 in the ABA response, the authors generated double or triple mutants from *vdac3* and tested the capacity of these lines to close their stomata upon ABA exposure (Fig. 1A). While the single mutants *vdac3*, *pyl1*, *pyl4*, *pyl8*, *pyr8*, *snrk2.2*, and *snrk2.3* did not show any difference in ABA-induced stomata closure, *pyr1* and *pyl2* lines showed to be partially insensitive to ABA by keeping their stomata not fully closed after treatment (Fig. 1C). Remarkably, crossing each of these single mutant lines with *vdac3*, every double mutant (*vdac3pyr1*, *vdac3pyl1*, *vdac3pyl4*, *vdac3pyl8*, *vdac3snrk2.2*, *vdac3snrk2.3*) was far less sensitive to ABA, observed as a failure to close their stomata after ABA treatments (Fig. 1D). The ABA-insensitive phenotype in the *vdac3* double mutants, also correlated with lower ROS levels after ABA exposure compared to the single mutants. Furthermore, the authors managed to revert the open stomata phenotype by directly treating the double mutants with hydrogen peroxide, indicating that ROS are an essential downstream component of the ABA signaling pathway modulated by VDAC3 (Fig. 1E).

This article unveils VDAC3 as a critical player in the redundancy control of ABA-mediated stomatal closure. The protein interacts with multiple PYLs, PYRs, and SnRK2s, forming a regulatory module crucial for ABA-dependent stomatal closure. The study suggests that VDAC3 acts as a scaffold protein in mitochondria, enhancing the efficiency of ABA-sensing complexes. Yet, future research will establish how conserved is this regulation in other plant species.

## Dive Curated Terms

The following phenotypic, genotypic, and functional terms are of significance to the work described in this paper:

PYRABACTIN CHEBI: CHEBI:73159

PYL4 Gramene: At2g38310
PYL4 Araport: At2g38310
